# Characterization of Printed Circuit Boards for Metal and Energy Recovery after Milling and Mechanical Separation

**DOI:** 10.3390/ma7064555

**Published:** 2014-06-16

**Authors:** Waldir A. Bizzo, Renata A. Figueiredo, Valdelis F. de Andrade

**Affiliations:** 1 Faculty of Mechanical Engineering, University of Campinas—UNICAMP, Campinas, SP 13083-970, Brazil; E-Mail: renatha345@yahoo.com.br; 2 The National Service of Commerce—SENAC, São Paulo, SP 04696-000, Brazil; E-Mail: valdelis.fandrade@sp.senac.br

**Keywords:** printed circuit boards, solid waste, heavy metals, energy recovery, metal recovery

## Abstract

The proper disposal of electrical and electronic waste is currently a concern of researchers and environmental managers not only because of the large volume of such waste generated, but also because of the heavy metals and toxic substances it contains. This study analyzed printed circuit boards (PCBs) from discarded computers to determine their metal content and characterized them as solid waste and fuel. The analysis showed that PCBs consist of approximately 26% metal, made up mainly of copper, lead, aluminum, iron and tin, as well as other heavy metals such as cadmium and nickel. Comparison with the results of other studies indicated that the concentration of precious metals (gold and silver) has declined over time. Analysis of the leachate revealed high concentrations of cadmium and lead, giving the residue the characteristics of hazardous waste. After milling the PCBs, we found that larger amounts of metal were concentrated in smaller fractions, while the lightest fraction, obtained by density separation, had a gross calorific value of approximately 11 MJ/kg, although with a high ash content. Milling followed by density separation proved potentially useful for recovery of metals and energy-rich fractions.

## 1. Introduction

The proper disposal of electrical and electronic waste is currently a concern for researchers and environmental managers not only because of the large volume of such waste generated, but also because of the heavy metals and toxic substances it contains. If this waste is not properly treated and disposed of, it can cause soil and groundwater contamination, resulting in risks to human health.

High concentrations of organic contaminants, including polybrominated diphenyl ethers and other carcinogens, have been found in soil at disposal sites, and a high concentration of heavy metals such as cadmium, lead, copper and zinc was observed in sediment samples from a river as a result of metal recovery being carried out using inappropriate techniques, such as open burning [1].

The electrical and electronic industry is one of the most innovative in terms of products, and this is reflected in the speed with which electronic devices such as cell phones and personal computers become obsolete, generating large amounts of electronic waste that must be treated.

In Japan, 40,000 tons of personal computers were discarded in 1999, and the corresponding figures for 2000 and 2002 were 50,000 and 80,000 tons, respectively, according to Hino *et al.* (2009) [2], demonstrating the significant annual increase in this type of waste. In China, 15 million household appliances, 5 million computers and 10 million cell phones were thrown away in 2004. Production of printed circuit boards is growing at 8.7% a year, and in 2003 China became the second largest producer in the world [3].

In Europe, 7 million tons of electronic waste is generated annually, and this figure is estimated to be increasing at 3%–5% a year [4].

In Brazil, 67 million mobile phones were marketed in 2011, compared with 59 million in 2012 [5]. The market for personal computers in Brazil stood at 15.5 million units in 2012, the same amount on the previous year. About 70% of these figures are accounted for by the official market, and it is estimated that 6 million handsets may have been imported irregularly without any control by the Brazilian government, making it difficult to monitor enforcement of the legislation requiring manufacturers to collect obsolete devices for recycling.

Practically all electronic devices contain printed circuit boards (PCBs), which are composed of three types of materials [6]:

- a non-conducting substrate or laminate;
- printed conducting tracks; and
- components mounted on the substrate.

The substrate is typically composed of glass fiber reinforced with epoxy resin or paper reinforced with phenolic resin, both with brominated flame retardants.

Three main types of materials can be retrieved from PCBs:

- recyclable metals, such as copper, aluminum, tin, lead and precious metals (gold, silver and platinum). Boards that have been produced recently may not have lead in their composition, but may contain other metals such as bismuth or silver;
- recyclable polymeric materials, from which energy can be recovered by combustion and incineration;
- ceramic materials, which can be reused or disposed of more appropriately if they are free of metals, polymers or other contaminants.

Metal-recovery processes and pretreatment processes studied by researchers include electrolytic processes, fusion and mechanical separation. The processes used to recycle PCBs can be divided into two categories according to the type of material recovered and the process used [6]: thermal processes (pyrometallurgical recovery) and non-thermal processes (electro/hydrometallurgical processes). Dismantling, milling, mechanical separation and pyrolysis are typical pretreatment processes.

A comparison of these processes, their main environmental impacts and related issues are shown in Table 1. It should be noted that a metal-recovery system may involve more than one of these processes, milling being an example of a process used in most systems for metal recovery from discarded PCBs.

**Table 1:** Comparison of the main processes for recovering metals from printed circuit boards (PCBs).

Types	Thermal Processes	Non-Thermal Processes
Characteristics	- Non-metallic materials cannot be recovered - High investment in equipment and installation, including air-pollution control systems - Economic efficiency not proven for low-grade wastes ^a^	- Health risks for the milling operators, because of the possibility of inhaling fiberglass particles and heavy metals - Strong irritating odor generated by phenolic resin during the milling process - Large investment in equipment for waste-water treatment
Environmental impacts	Generation of gaseous pollutants, including dioxins and lead fumes	- High water utilization and waste-water generation, with acidic residues - Noise pollution due to grinding equipment - Generation of solid waste

^a^ Adapted from Xiang *et al.* (2007) [7] and Lee *et al.* (2004) [8].

In Brazil, one recycling plant is known to recover copper from PCBs. This small-scale plant uses a sequence of processes including milling, burning, acidic treatment and galvanic deposition. The other recycling plants identified as part of this study do not recover metals but only mill PCBs, which are then sent to other countries for the metals to be recovered. However, most discarded PCBs are usually disposed of in sanitary landfills, and none of the metals in them are recovered.

Of the various pretreatment processes for recovering metals from PCBs, mechanical separation is one of the more promising because it has certain technological and environmental advantages: it neither produces a chemical change in the components nor uses water as a processing medium, thus avoiding the generation of liquid effluents [9]. All metal-recovery processes, however, involve some degree of energy consumption, which must be properly evaluated.

Mechanical separation involves milling and separating particles according to their size by screening or another process [10]. While such processes are unlikely to allow metals to be recovered with the purity needed for immediate recycling, they can help increase the concentration of metals, facilitating final recovery operations.

Separation of the polymeric and metal fractions produces a material that can be incinerated to recover energy. Combustion of the polymeric fraction also generates ashes because of the mineral content of PCBs.

The aims of this study were to:

- characterize the chemical composition of PCBs from discarded computers and compare the results with the findings of others studies, with an emphasis on metals recovery;
- characterize the combustible fraction from discarded PCBs, focusing on energy recovery;
- investigate the possibility of increasing the concentration of metals in PCB waste by milling and separation based on particle size; and
- characterize PCBs in terms of the hazard they represent as contaminants.

## 2. Results and Discussion

### 2.1. Characterization as Solid Waste

The physical and chemical results for the leachate and the criteria for hazard characterization of the residue, with the maximum permitted concentration of each element defined by the EPA in CFR Title 40 [11], are shown in Table 2.

**Table 2:** Chemical analysis of leachate from milled PCBs.

Substance	Results of this study (mg/L)	Maximum permitted concentration according to EPA legislation (mg/L) *
Arsenic	ND	5.0
Cadmium	22.0	0.5
Lead	133	5.0
Barium	1.5	100.0
Chromium	0.05	5.0
Selenium	ND	1.0
Silver	0.02	5.0
Mercury	ND	0.2
Fluoride	ND	–

ND = undetected; * Source: EPA—CFR Title 40.

As the cadmium and lead contents in the leachate exceeded the limits allowed under EPA legislation, the PCB residue can be classified as hazardous waste. Many countries, including Brazil, use criteria and legislation similar to that contained in CFR Title 40 to characterize solid waste as hazardous [12].

### 2.2. Metal Concentration

The results of the chemical analysis of samples from full boards (*i.e.*, components and laminates) are shown in Table 3, which also shows the results reported by other authors. The table shows that the metal content varies from 20% to 40% by weight and averages 30%. The average metal content for the samples analyzed in this work was 27%. This variation can be explained by the wide range of board types used, the different characterization methods used by the various authors and the change in the composition of PCBs over the years. The predominant metals are copper, aluminum, iron, tin and lead.

**Table 3:** Chemical composition of PCBs in the present study and values reported by other authors.

Metal content	a	b	c	d	e	f	g	h	i	j	k	l	m	This study
Cu (%)	19	20	22	12.5	26.8	15.6	19.66	28.7	27.6	14.6	12.58	19.19	28	14.2
Al (%)	4.1	2	–	2.04	4.7	–	2.88	1.7	–	–	2.38	7.06	2.6	–
Pb (%)	1.9	2	1.55	2.7	–	1.35	3.93	1.3	–	2.96	2.44	1.01	–	2.50
Zn (%)	0.8	1	–	0.08	1.5	0.16	2.10	–	2.7	–	–	0.73	–	0.18
Ni (%)	0.8	2	0.32	0.7	0.47	0.28	0.38	–	0.3	1.65	0.39	5.35	0.26	0.41
Fe (%)	3.6	8	3.6	0.6	5.3	1.4	11.47	0.6	2.9	4.79	3.24	3.56	0.08	3.08
Sn (%)	1.1	4	2.6	4.0	1.0	3.24	3.68	3.8	–	5.62	1.41	2.03	–	4.79
Sb (%)	–	–	–	–	0.06	–	–	–	–	–	–	–	–	0.05
Cr (%)	–	–	–	–	–	–	0.005	–	–	0.356	–	–	–	–
Na (%)	–	–	–	–	–	–	–	–	–	–	–	–	–	0.48
Ca (%)	–	–	–	–	–	–	1.13	–	1.4	–	–	–	–	1.69
Ag (ppm)	5210	2000	–	300	3300	1240	500	79	–	450	–	100	135	317
Au (ppm)	1120	1000	350	-	80	420	300	68	–	205	–	70	29	142
Pt (ppm)	–	–	–	–	–	–	–	0	–	–	–	–	–	–
Cd (ppm)	–	–	–	–	–	–	–	–	–	–	–	–	–	1183
K (ppm)	–	–	–	–	–	–	–	–	–	–	–	–	–	180
In (ppm)	–	–	–	–	–	–	500	–	–	–	–	–	–	–
Mn (ppm)	–	–	–	–	–	–	9700	–	4000	–	–	–	–	81
Se (ppm)	–	–	–	–	–	–	–	–	–	–	–	–	–	21
As (ppm)	–	–	–	–	–	–	–	–	–	–	–	–	–	11
Mg (ppm)	–	–	–	500	–	–	1000	–	–	–	–	–	–	–
Pd (ppm)	–	50	–	–	–	–	–	33	–	220	–	–	–	–
Co (ppm)	–	–	–	–	–	–	300	–	–	–	–	400	–	–
Ti (ppm)	–	–	–	–	–	–	–	–	–	–	–	400	–	–
**Total Metals (%)**	**31.9**	**39.3**	**30.1**	**22.6**	**40.2**	**22.2**	**46.5**	**36.1**	**35.3**	**30.1**	**22.5**	**39.1**	**31.1**	**27.6**

(a) Feldman (1993) [13]; (b) Menetti *et al.* (1995) [14]; (c) Iji *et al.* (1997) [15]; (d) Veit *et al.* (2002) [16]; (e) Zhao *et al.* (2004) [17]; (f) Kim *et al.* (2004) [18]; (g) Wang *et al.* (2005) [19]; (h) Creamer *et al.* (2006) [20]; (i) Marco *et al.* (2008) [21]; (j) Hino *et al.* (2009) [2]; (k) Das *et al.* (2009) [22]; (l) Yoo *et al.* (2009) [23]; (m) Oliveira *et al.* (2010) [4].

The content of precious metals such as silver and gold in PCBs has fallen in recent years. While gold contents above 1000 ppm were reported in 1993 and 1995, values reported since then are all below 1000 ppm, most values being below 100 ppm.

Table 4 shows the amounts of various metals found in nature as mineral deposits and the range in content of the same metals in PCBs. It can be seen that four of these metals (copper, tin, lead and nickel) are found in discarded PCBs in concentrations higher than or equal to those in natural deposits, showing the importance of electronic waste as a possible source of raw material and the incentive that exists to develop processes to recover these metals from discarded PCBs.

**Table 4:** Metal content of ores and PCBs.

Metal	Ores (%) ^a^	PCBs (%) ^b^
Copper	0.5–3.0	12.0–29.0
Zinc	1.7–6.4	0.1–2.7
Tin	0.2–0.85	1.1–4.8
Lead	0.3–7.5	1.3–3.9
Iron	30–60	0.1–11.4
Nickel	0.7–2.0	0.3–1.6
Gold	0.0005	0.0029–0.112
Silver	0.0005	0.01–0.52

Source: ^a^ Veit *et al.* (2002) [16], Ayres (1997) [24]; ^b^ range of results reported by various authors as shown in Table 3.

**Figure 1:**
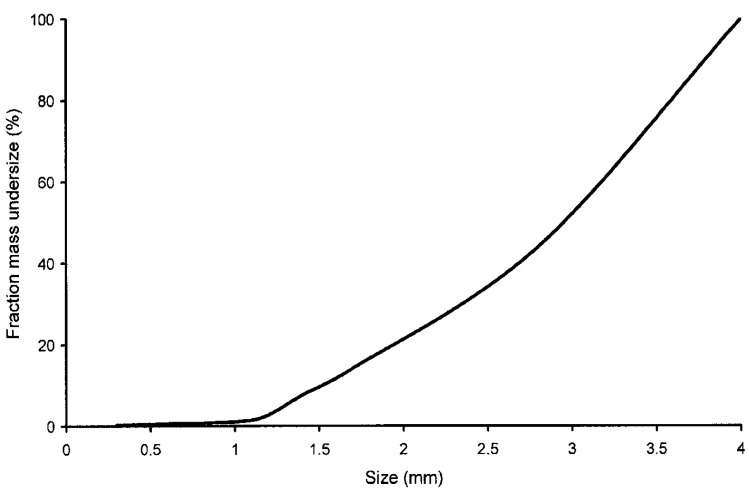
Particle-size distribution after milling.

**Figure 2:**
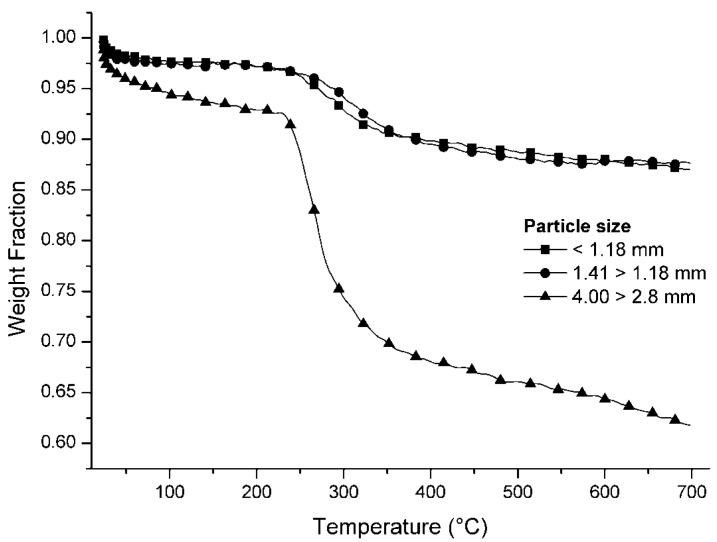
Thermogravimetric analysis of ground PCBs separated by particle size.

The particle-size distribution after milling is shown in Figure 1, and the results of thermogravimetric analysis of three particle-size fractions are shown in Figure 2. Peak weight loss occurs between 230 °C and 240 °C as a result of pyrolysis of the light fraction of the PCBs, which usually consist of thermoplastic polymers (substrate) and phenolic resin. This temperature is lower than the peak evolution temperatures of volatiles in most polymers typically found in municipal waste, which are in the range 400–500 °C, according to Heikkinen *et al.* (2004) [25].

Total mass loss due to pyrolysis up to 700 °C was 27%–36% in the fraction with particle sizes between 4.00 and 2.8 mm and 11%–16% in the fraction with particle sizes below 1.18 mm. The latter fraction had an inorganic material content of 84%–89%, compared with a figure of 64%–73% for the larger fractions.

As most metals do not suffer pyrolysis or devolatilization up to this temperature, it would appear that the metals were more concentrated in the fractions with the smallest particle size after milling. Milling is therefore an effective method for separating metals, and the results obtained using this technique can be improved by successive sieving operations. Milling and sieving are usually simple, low-cost operations with minimal environmental impact.

### 2.3. Characterization of PCBs in Terms of Calorific Value

When the PCB samples were separated by density using a liquid with a density of 2.89 g∙cm^−3^, 51% of the total sample was recovered in the heavy fraction. The light fraction consisted mainly of polymer and glass fiber and represented 49% of the mass of the boards. The results of ultimate analysis of the light fraction are C: 67.8% ± 4.0%, H: 5.6% ± 0.5% and N: 2.2% ± 0.3%, dry and ash free. The results of the proximate analysis and the GCV (gross calorific value) of the light fraction and total fraction are shown in Table 5.

**Table 5:** Proximate analysis and GCV of milled PCBs.

Samples	Moisture (% wet basis)	Volatiles (% dry basis)	Fixed carbon (% dry basis)	Ashes (% dry basis)	Gross calorific value (MJ/kg)
Light fraction	0.87	39.85	2.38	57.78	11.63
Total fraction	0.37	16.70	0.99	82.27	4.88

It can be seen from Table 6 that the light fraction has a higher GCV, higher volatile content and lower ash content than the total fraction. Its elementary composition, expressed on a dry ash-free basis, includes C and H in a high C/H mass ratio approximately equal to typical values for polymers normally used in PCBs. These results illustrate the advantages of gravimetric separation of milled PCB particles, as this technique ensures that the light fraction has characteristics typical of residual fuels. Although its GCV (11.6 MJ/kg) is slightly greater than that of urban waste (approximately 8.3 MJ/kg [26]), the light fraction still has a high ash content (58%). The GCV is slightly lower than that of Brazilian mineral coals, for which the corresponding figure is about 12–14 MJ/kg and the ash content about 50%–59% [27,28]. It should be stressed that the diversity of shapes and sizes in the light fraction makes it difficult to obtain a representative sample and so ensure a more reliable analysis of the elements it contains.

## 3. Materials and Methods

**Figure 3:**
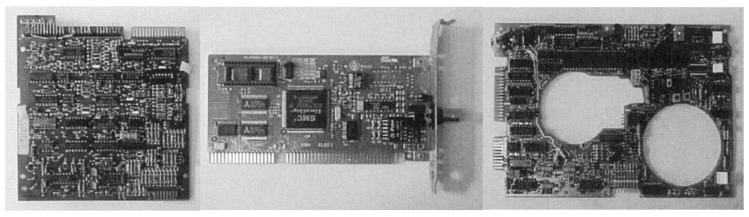
PCBs used in the study, without cables, wires and connectors.

The PCBs used in this study were collected from scrap stored at the Unicamp Faculty of Mechanical Engineering (FEM). Approximately 12 kg of PCBs from computers of various types (XT, 486 and Pentium) and ages were used (Figure 3). After the cables, wires and connectors had been manually removed, the boards, containing all their remaining components (chips, condensers, resistors), were ground in a cutting mill with a 9 mm grid.

**Figure 4:**
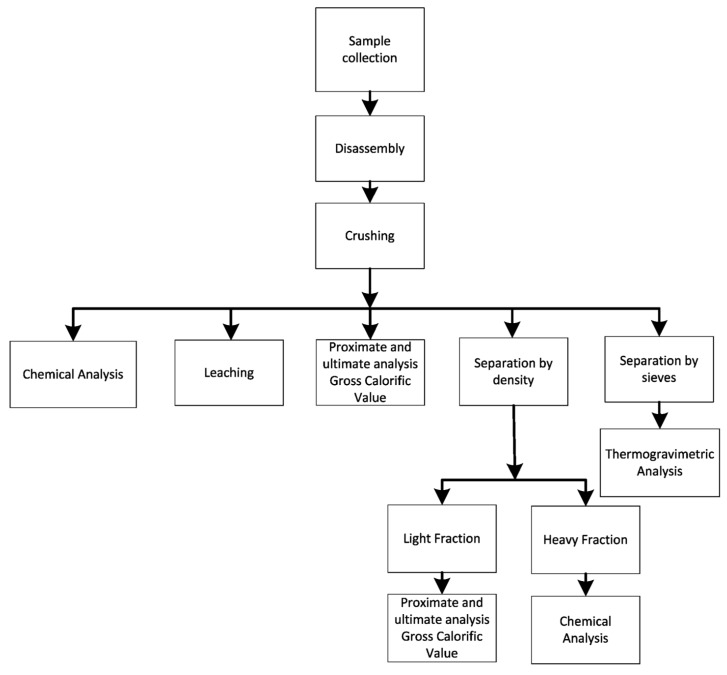
Steps involved in the characterization of discarded PCBs.

Five samples of the milled material were collected. Samples were obtained using the quartering method. Three samples were used for chemical analyses, leaching tests and proximate and ultimate analysis, as well as to determine the gross calorific value (GCV). A fourth sample was separated by particle size using sieves, and then characterized by thermogravimetric analysis for each fraction size. The fifth sample was separated by density difference into a light fraction (fiber, polymers and ceramic materials) and heavy fraction (metals and ceramic materials) using bromoform (CHBr_3_, density = 2.89 g·cm^−3^). The heavier particles decanted, while the lighter fraction floated on the surface [29]. The heavy fraction was analyzed to determine its chemical composition, while the light fraction was subjected to proximate and ultimate analysis, and its GCV was determined. A flowchart of the steps involved is shown in Figure 4.

The sample was prepared for chemical analysis by digestion with aqua regia [30]. After digestion the sample was filtered, and dilutions were made according to the element to be analyzed and the analysis method used: atomic absorption spectrometry (AAS), inductively couple plasma (ICP). Leaching analyses were performed according to the Environmental Protection Agency (EPA) SW-846 test method [31]. Thermogravimetric analyses were performed on a thermogravimetric balance in an inert atmosphere using nitrogen (100 mL∙min^−^^1^). The samples used in the analyses represented three particle size ranges: between 4.00 and 2.8 mm, between 1.41 and 1.18 mm and smaller than 1.18 mm. Sample mass was 5.0 ± 0.5 g, and heating rate was 10 °C∙min^−^^1^ up to a temperature of 700 °C. Proximate and ultimate analyses were performed according to ASTM D-3173-87 (moisture and fixed carbon) [32], D-3175-89a (volatile material) [33] and D 3174-93 (ash content) [34]. Calorific value was determined in a bomb calorimeter according to ASTM D-2015 [35].

## 4. Conclusions

This study analyzed PCBs from discarded computers by determining their metal content and characterizing them as solid waste and fuel.

Analysis of the leachate indicates that PCBs can potentially cause significant contamination of soil, groundwater and surface water by heavy metals (lead and cadmium) if the boards and components are not correctly disposed of. The results suggest that according to CFR Title 40, PCBs should be classified as hazardous waste.

Based on chemical analysis and comparison of our results with those reported by other authors, PCBs can be considered raw material for metal recovery, as their metal content is greater than or similar to that of naturally occurring ores.

In the PCBs analyzed, the metal present in the greatest quantities was copper, and significant quantities of lead, iron and tin were also observed. Precious metals such as gold and silver were present. However, concentrations of these elements have decreased in recent years.

For the sample milled and separated by particle size with sieves, the fraction with particle size <1.18 mm had a higher inorganic material content (84%–89%) than the fraction with larger particle size (>1.18 mm) (64%–73%).

For the sample milled and separated by density, the light fraction (<2.89 g∙cm^−^^3^) had average volatile content (39.85%) and a GCV of 11.63 MJ∙kg^−^^1^, potentially justifying the separation of discarded PCBs into fractions so that the light fraction can, despite its high ash content (58%), be incinerated for energy recovery. Gasification or pyrolysis can also be used for energy recovery or production of raw materials.

This study has shown that mechanical pre-treatment of discarded PCBs can be advantageous. The prior separation of metals by milling and sieving has the potential to improve the performance of subsequent processes for metal recovery and energy recovery by incineration as it increases the inorganic material content in smaller, particle-size fractions.
